# Gut Microbiota Manipulation to Mitigate the Detrimental Effects of Environmental Pollutants

**DOI:** 10.3390/toxics9060127

**Published:** 2021-06-01

**Authors:** Lianguo Chen

**Affiliations:** State Key Laboratory of Freshwater Ecology and Biotechnology, Institute of Hydrobiology, Chinese Academy of Sciences, Wuhan 430072, China; lchenam@ihb.ac.cn

The ecotoxicology and human health risks of environmental pollutants are creating global concern, especially in the context of the prevalent and severe contamination of environmental abiotic and biotic compartments. It has been confirmed that long-term exposure to the toxication of pollutants potentially increases the propensity for developing metabolic and carcinogenic diseases. Notably, contamination of water and food facilitates the exposure to environmental pollutants via dietary routes, which can directly reach the gut ecosystem and come in contact with the microflora commensally residing there. As the first line of defense against the ingestion and toxicity of environmental xenobiotics, the gut-microbiota system is commonly targeted and driven to dysbiosis by a variety of pollutants in spite of their distinct physicochemical nature, including persistent organic pollutants, heavy metals and food additives. In recent years, mounting evidence has verified the fundamental involvement of gut microbiota in the maintenance and regulation of overall host health. Therefore, disturbances in the gut microbial community with respect to the composition, abundance, diversity and metabolism will subsequently lead to the compromise of host physiological activities and fitness, which underlies the pathogenesis of various diseases (e.g., diabetes, obesity, inflammation, and colitis).

Considering the targeted effects of environmental pollutants on the gut and the vital importance of gut microbiota to host health, utilization of dietary intervention to restore the functions of the gut microbial population and the integrity of the gut epithelial barrier appears to be a simple but feasible approach to fighting the detrimental effects of environmental pollutants. Currently, probiotic bacteria and prebiotic substances have been widely tested for their potential to improve the development, growth and health of aquaculture animals and human beings. However, the capacity and mechanism of probiotics and prebiotics to attenuate or mitigate the toxicities of pollutants remain largely elusive from a toxicological perspective. In particular, the tremendous cost associated with the elimination of persistent toxic substances from the environment encourages the development of in situ remediation of animal and human health issues related to the lasting stress of environmental pollutants, among which dietary supplementation of probiotics and prebiotics is promising.

Probiotic bacteria refer to certain live microorganisms that are able to have beneficial effects on the metabolism and health of hosts once consumed, generally by shaping the gut microbiome. Regarding the toxicities of environmental pollutants, probiotic administration has also been shown to yield inhibitory activities. For example, treatments with *Lactobacillus plantarum* CCFM8610 efficiently sequester the heavy metal cadmium, and thus, reduce the bioaccumulation. In addition, *L. plantarum* CCFM8610 can protect the intestinal barrier and prevent mice from developing cadmium-induced inflammatory symptoms [1]. Oral supplementation of *L. plantarum* TW1-1 alleviates the endocrine disruption and testicular toxicities of diethylhexylphthalate in male mice whilst shifting the exposed gut microbiota to the control structure and reducing the hyper-permeability of the intestinal barrier induced by diethylhexylphthalate pollutant [2]. In addition, dietary administration of probiotic *L. rhamnosus* significantly mitigates the accumulation of cholesterol in the blood and antagonizes the disturbance of bile acid metabolism in zebrafish due to the chronic exposure of perfluorobutanesulfonate, an aquatic pollutant of emerging concern [3].

Prebiotics are defined as non-digestible food ingredients that can be fermented by the gut microbiota, and thus, selectively promote the growth and/or activity of certain gut bacterial species in an attempt to confer benefits upon the host’s well-being and health. The principal prebiotics that have gained wide acceptance include a battery of dietary fibers, including fructooligosaccharides, mannanoligosaccharides, galactooligosaccharides, and inulin, which also show good potency for alleviating the hazardous effects of xenobiotics. Consumption of saccharin and sucralose, two representatives of noncaloric artificial sweeteners, was found to disrupt the gut microbiota community, increase intestinal permeability, induce systemic inflammation and cause nonalcoholic fatty liver disease (NAFLD) in mice, while fructooligosaccharide supplementation significantly stimulates *Akkermansia muciniphila* reproduction in sucralose-consuming mice, consequently ameliorating NAFLD [4]. In addition, inulin feeding of hyperlipidemic mice restores gut microbiota, decreases aortic root lesions, attenuates glucose metabolism disruption and reduces hepatic lipid accumulation after exposure to a dioxin-like pollutant PCB126 [5]. 

Therefore, functional dietary intervention represents a valid means for the prevention and control of various diseases resulting from xenobiotic challenges. Although probiotic and prebiotic applications have been introduced in the aquaculture industry and for human health improvement, whether these functional ingredients can interact with and inhibit the innate toxicity of environmental pollutants still lacks sufficient investigation. More combined exposures are expected in the future to elucidate the beneficial mode of action of probiotics and prebiotics against the toxicities of environmental xenobiotics, thereby highlighting their protective value. Considering the misuse and the resulting ubiquitous pollution of antibiotics, the development of a probiotic and prebiotic formulation will provide an environmentally-friendly alternative choice to enhance aquaculture output and improve human health.
